# Supplementary material for: Association between blood pressure categories and cardiovascular disease mortality in China
Source: PLoS One. 2021 Jul 30;16(7):e0255373. doi: 10.1371/journal.pone.0255373 (PMC8323908; doi:10.1371/journal.pone.0255373)
Supplement: S1 Fig — (DOCX) [file pone.0255373.s002.docx]

**
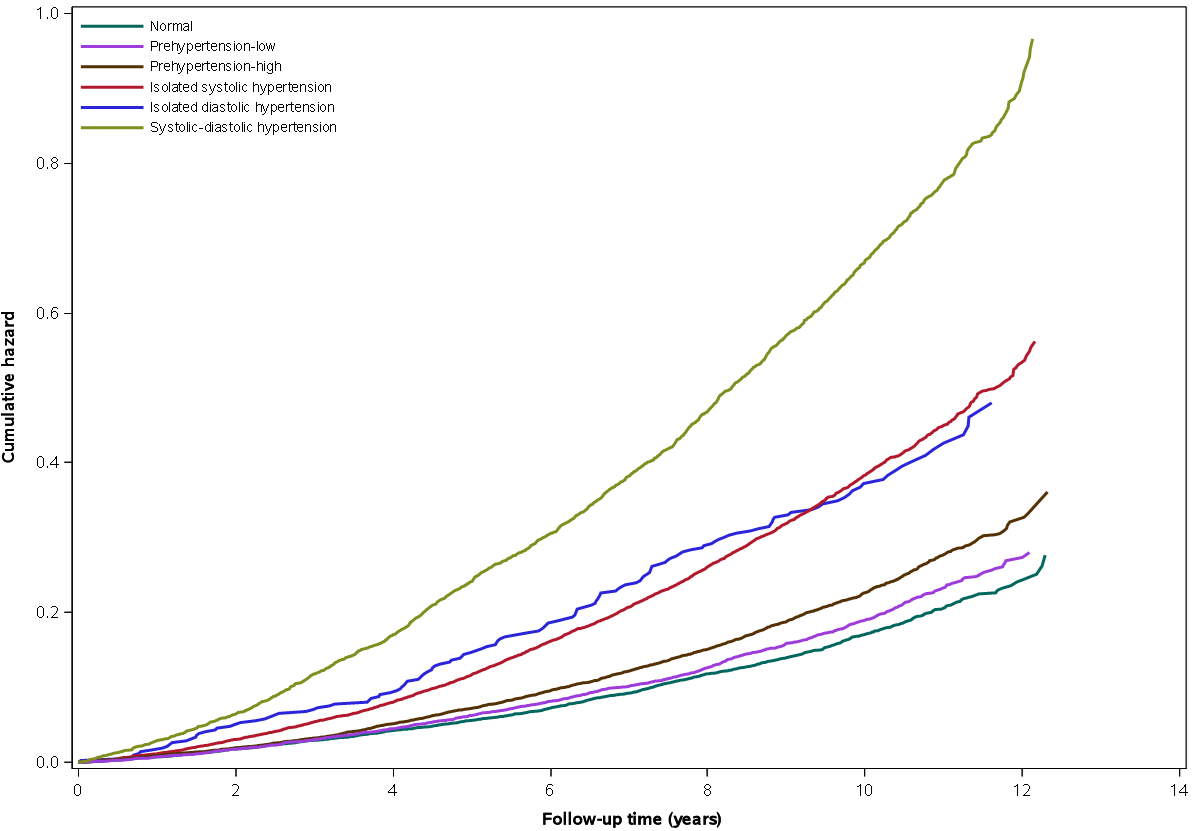
**

**S1 Fig. Nelson-Aalen cumulative hazard for cardiovascular diseases according to the blood pressure categories**

Adjusted for age at risk (in 5-year intervals), sex and survey sites, education level, marital status, smoking status, alcohol consumption, intake frequencies of vegetables, fruits, and red meat, physical activity, body mass index, survey season, heart rate, diabetes at baseline, family history of cardiovascular disease (i.e., heart attack and stroke).
