# Supplementary material for: Association between blood pressure categories and cardiovascular disease mortality in China
Source: PLoS One. 2021 Jul 30;16(7):e0255373. doi: 10.1371/journal.pone.0255373 (PMC8323908; doi:10.1371/journal.pone.0255373)
Supplement: S2 Fig — (DOCX) [file pone.0255373.s003.docx]

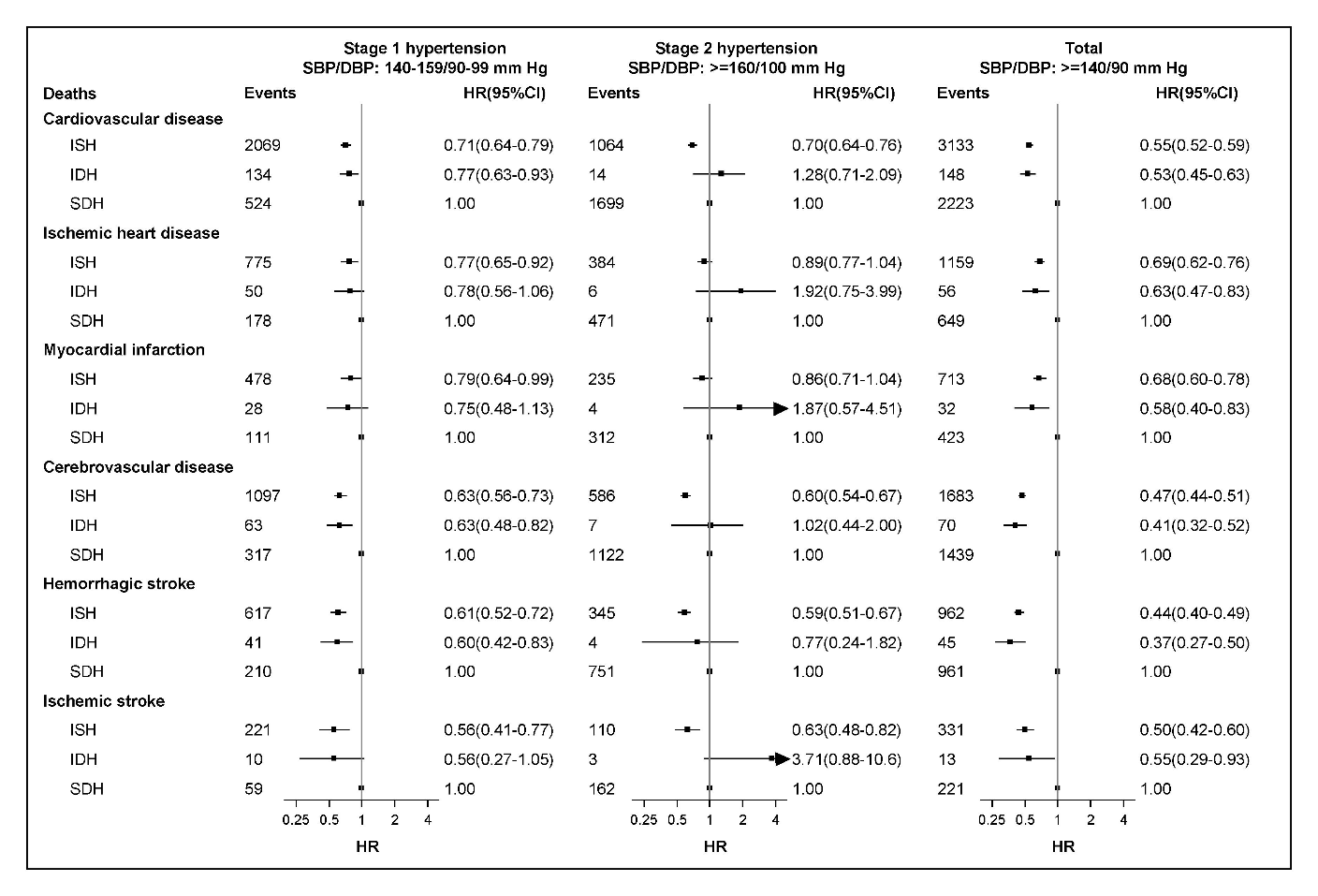


**S2 Fig. Associations of ISH, IDH, and SDH with Mortality from CVDs and its Major Subtypes in Stage 1 Hypertension, Stage 2 Hypertension and Total Hypertension**

Abbreviations: ISH, isolated systolic hypertension; IDH, isolated diastolic hypertension; SDH, systolic-diastolic hypertension; CVDs, cardiovascular diseases.

Stage 1 and stage 2 hypertension were defined based on the Seventh Report of the Joint National Committee on the Prevention, Detection, Evaluation, and Treatment of High Blood Pressure (JNC-7).

Multi-adjusted hazard ratios were adjusted for age, education level, marital status, smoking status, alcohol consumption, intake frequencies of vegetables, fruits, and red meat, physical activity, body mass index, survey season, heart rate, diabetes at baseline, family history of heart attack, stroke (only adjusted for in corresponding analysis of cause-specific mortality) and were stratified according to age at risk (in 5-year intervals), sex and survey sites. Data markers represent the point estimate of hazard ratio. Error bars represent 95% confidence interval. Black arrows represent the confidence intervals exceed the x-axis value.
