# Supplementary material for: Association between blood pressure categories and cardiovascular disease mortality in China
Source: PLoS One. 2021 Jul 30;16(7):e0255373. doi: 10.1371/journal.pone.0255373 (PMC8323908; doi:10.1371/journal.pone.0255373)
Supplement: S1 Table — (DOCX) [file pone.0255373.s004.docx]

**S1 Table. Baseline characteristics of the study population by baseline BP categories**

| **Characteristic** | **Normal (SBP <120 and DBP <80 mmHg)** | **Prehypertension-low (SBP 120-129 and DBP <80 mmHg)** | **Prehypertension-high (SBP 130-139 and/or DBP 80-89 mmHg)** | **Hypertension** | | |
| --- | --- | --- | --- | --- | --- | --- |
|  |  |  |  | **ISH (SBP ≥140 and DBP <90 mmHg)** | **IDH (SBP <140 and DBP ≥90 mmHg)** | **SDH (SBP ≥140 and DBP ≥90 mmHg)** |
| Total, No. | 144 765 | 70 130 | 107 960 | 60 708 | 8387 | 39 027 |
| Age, mean (SD), y | 47.1 (9.4) | 49.8 (10.4) | 49.8 (9.9) | 57.3 (10.1) | 47.3 (8.2) | 52.2 (9.6) |
| Female, No. (%) | 96 918 (66.9) | 39 937 (56.9) | 58 329 (54.0) | 35 134 (57.9) | 3794 (45.2) | 18 784 (48.1) |
| Education level, No. (%) |  |  |  |  |  |  |
| No formal education or primary school | 59 294 (41.0) | 35 191 (50.2) | 53 136 (49.2) | 40 644 (66.9) | 3246 (38.7) | 21 588 (55.3) |
| Middle or high school | 73 853 (51.0) | 31 459 (44.9) | 49 166 (45.5) | 18 249 (30.1) | 4442 (53.0) | 15 814 (40.5) |
| College or higher | 11 618 (8.0) | 3480 (5.0) | 5658 (5.2) | 1815 (3.0) | 699 (8.3) | 1625 (4.2) |
| Rural area, No. (%) | 73840(51.0) | 42291(60.3) | 65486(60.7) | 37856(62.4) | 4324(51.6) | 24396(62.5) |
| Married, No. (%) | 134 038 (92.6) | 64 446 (91.9) | 99 571 (92.2) | 52 636 (86.7) | 7849 (93.6) | 35 419 (90.8) |
| Regular smoking, No. (%) | 33 951 (23.5) | 20 331 (29.0) | 32 518 (30.1) | 16 082 (26.5) | 2917 (34.8) | 13 142 (33.7) |
| Male | 31 785 (66.4) | 19 323 (64.0) | 31 197 (62.9) | 15147 (59.2) | 2852 (62.1) | 12689 (62.7) |
| Female | 2166(2.2) | 1008(2.5) | 1321(2.3) | 935(2.7) | 65(1.7) | 453(2.4) |
| Regular alcohol intake, No. (%) | 15 732 (10.9) | 10 150 (14.5) | 19 655 (18.2) | 9628 (15.9) | 2196 (26.2) | 9724 (24.9) |
| Male | 13 635 (28.5) | 9330 (30.9) | 18 320 (36.9) | 8875 (34.7) | 2085 (45.4) | 9239 (45.6) |
| Female | 2097 (2.2) | 820 (2.1) | 1335 (2.3) | 753 (2.1) | 111 (2.9) | 485 (2.6) |
| Average weekly consumption, mean (D), day/week ^a^ | | |  |  |  |  |
| Fresh vegetables | 6.83(0.80) | 6.83(0.79) | 6.84(0.77) | 6.82(0.82) | 6.83(0.82) | 6.82(0.84) |
| Fresh fruits | 2.71(2.72) | 2.30(2.57) | 2.32(2.58) | 1.97(2.46) | 2.57(2.69) | 2.02(2.47) |
| Red meat | 3.88(2.63) | 3.62(2.60) | 3.66(2.60) | 3.36(2.58) | 3.94(2.64) | 3.47(2.61) |
| Postmenopausal, No. (%) ^b^ | 33 002 (34.1) | 18 865 (47.2) | 27 871 (47.8) | 26 430 (75.2) | 1447 (38.1) | 11 023 (58.7) |
| Physical activity, mean (SD), MET- hr/day | 22.4 (13.6) | 22.7 (14.1) | 22.6 (14.1) | 19.7 (13.9) | 22.9 (14.1) | 22.0 (14.3) |
| Heart rate, mean (SD), bpm | 77.0 (10.5) | 77.9 (11.1) | 79.7 (11.7) | 79.2 (12.6) | 82.8 (12.6) | 82.7 (12.9) |
| Body mass index, mean (SD), kg/m^2^ | 22.5 (2.9) | 23.2 (3.1) | 23.8 (3.2) | 23.9 (3.5) | 24.6 (3.4) | 24.7 (3.5) |
| Diabetes at baseline, No. (%) | 3574 (2.5) | 2635 (3.8) | 4847 (4.5) | 5177 (8.5) | 366 (4.4) | 2409 (6.2) |
| Family medical history, No. (%) | 25 525 (17.6) | 12 555 (17.9) | 21 193 (19.6) | 11 662 (19.2) | 2012 (24.0) | 9347 (24.0) |
| Heart attack | 4707 (3.3) | 2078 (3.0) | 3314 (3.1) | 1613 (2.7) | 338 (4.0) | 1329 (3.4) |
| Stroke | 21 875 (15.1) | 10 918 (15.6) | 18 684 (17.3) | 10 445 (17.2) | 1772 (21.1) | 8390 (21.5) |
| SBP, mean (SD), mmHg | 109.2 (7.4) | 124.4 (2.8) | 129.9 (6.8) | 151.3 (10.7) | 133.1 (5.2) | 162.4 (17.7) |
| DBP, mean (SD), mmHg | 68.1 (6.2) | 72.3 (5.2) | 80.2 (5.9) | 80.6 (6.6) | 92.6 (2.7) | 97.5 (6.9) |

Abbreviations: BP, blood pressure; SD, standard deviation; MET, metabolic equivalent of task; bpm, beat per minute.

Data was presented as mean (standard deviation) for continuous variables or number (proportion, %) for categorical variables.

^a^ Average weekly consumptions of red meat, fresh vegetables, and fruits were calculated by assigning participants to the midpoint of their consumption category.

^b^ Only for female.
