# Supplementary material for: Association between blood pressure categories and cardiovascular disease mortality in China
Source: PLoS One. 2021 Jul 30;16(7):e0255373. doi: 10.1371/journal.pone.0255373 (PMC8323908; doi:10.1371/journal.pone.0255373)
Supplement: S2 Table — (DOCX) [file pone.0255373.s005.docx]

**S2 Table. Associations of blood pressure categories with cardiovascular diseases mortality among 430 977 participants ^a^**

| **Cause of death** | **Normal** | **Prehypertension ^b^** | **Hypertension** | | |
| --- | --- | --- | --- | --- | --- |
|  |  |  | **ISH** | **IDH** | **SDH** |
| No. of participants | 144 765 | 178 090 | 60 708 | 8387 | 39 027 |
| No. of person-years | 1 462 055 | 1 788 282 | 593 913 | 85 056 | 383 785 |
| Cardiovascular disease |  |  |  |  |  |
| No. of deaths | 1330 | 2826 | 3133 | 148 | 2223 |
| Incidence rate (no./1,000 person-y) | 0.91 | 1.58 | 5.28 | 1.74 | 5.79 |
| HR (95%CI) | 1.00 | 1.23 (1.15-1.31) | 2.04 (1.90-2.18) | 2.19 (1.84-2.60) | 3.79 (3.53-4.08) |
| Ischemic heart disease |  |  |  |  |  |
| No. of deaths | 586 | 1114 | 1159 | 56 | 649 |
| Incidence rate (no./1,000 person-y) | 0.40 | 0.62 | 1.95 | 0.66 | 1.69 |
| HR (95%CI) | 1.00 | 1.08 (0.98-1.20) | 1.66 (1.49-1.85) | 1.76 (1.32-2.30) | 2.48 (2.20-2.79) |
| Myocardial infarction |  |  |  |  |  |
| No. of deaths | 378 | 702 | 713 | 32 | 423 |
| Incidence rate (no./1,000 person-y) | 0.26 | 0.39 | 1.20 | 0.38 | 1.10 |
| HR (95%CI) | 1.00 | 1.06 (0.93-1.20) | 1.64 (1.44-1.88) | 1.57 (1.07-2.23) | 2.44 (2.11-2.83) |
| Cerebrovascular disease |  |  |  |  |  |
| No. of deaths | 577 | 1399 | 1683 | 70 | 1439 |
| Incidence rate (no./1,000 person-y) | 0.39 | 0.78 | 2.83 | 0.82 | 3.75 |
| HR (95%CI) | 1.00 | 1.39 (1.26-1.53) | 2.51 (2.27-2.78) | 2.50 (1.93-3.19) | 5.58 (5.04-6.18) |
| Hemorrhagic stroke |  |  |  |  |  |
| No. of deaths | 315 | 809 | 962 | 45 | 961 |
| Incidence rate (no./1,000 person-y) | 0.22 | 0.45 | 1.62 | 0.53 | 2.50 |
| HR (95%CI) | 1.00 | 1.51 (1.32-1.72) | 2.89 (2.53-3.31) | 2.85 (2.05-3.86) | 6.88 (6.02-7.88) |
| Ischemic stroke |  |  |  |  |  |
| No. of deaths | 126 | 274 | 331 | 13 | 221 |
| Incidence rate (no./1,000 person-y) | 0.09 | 0.15 | 0.56 | 0.15 | 0.58 |
| HR (95%CI) | 1.00 | 1.20 (0.97-1.49) | 1.98 (1.60-2.47) | 2.04 (1.10-3.49) | 3.74 (2.99-4.69) |

Abbreviations: ISH, isolated systolic hypertension; IDH, isolated diastolic hypertension; SDH, systolic-diastolic hypertension; HR, hazard ratios; CI, confidence interval.

^a^ Multi-adjusted hazard ratios were adjusted for age, education level, marital status, smoking status, alcohol consumption, intake frequencies of vegetables, fruits, and red meat, physical activity, body mass index, survey season, heart rate, diabetes at baseline, family history of cardiovascular disease and were stratified according to five-year age group, sex, and survey sites.

^b^ Prehypertension was defined as SBP/DBP: 120-139/80-89mmHg.
