# Supplementary material for: Association between blood pressure categories and cardiovascular disease mortality in China
Source: PLoS One. 2021 Jul 30;16(7):e0255373. doi: 10.1371/journal.pone.0255373 (PMC8323908; doi:10.1371/journal.pone.0255373)
Supplement: S3 Table — Values are hazard ratios (95% confidence interval). (DOCX) [file pone.0255373.s006.docx]

**S3 Table. Associations of blood pressure categories with mortality from cardiovascular diseases and its major subtypes. Values are hazard ratios (95% confidence interval) ^a^**

| **Cause of death** | **Prehypertension-low** | **Prehypertension-high** | **Hypertension** | | |
| --- | --- | --- | --- | --- | --- |
|  |  |  | **ISH** | **IDH** | **SDH** |
| Cardiovascular disease |  |  |  |  |  |
| Model 1 | 1.06 (0.97-1.15) | 1.27 (1.18-1.37) | 1.96 (1.83-2.09) | 2.14 (1.80-2.53) | 3.69 (3.45-3.96) |
| Model 2 | 1.08 (0.99-1.17) | 1.30 (1.21-1.40) | 2.00 (1.87-2.14) | 2.18 (1.83-2.58) | 3.72 (3.47-3.99) |
| Model 3 | 1.10 (1.01-1.19) | 1.32 (1.23-1.42) | 2.04 (1.91-2.19) | 2.20 (1.85-2.61) | 3.81 (3.54-4.09) |
| Ischemic heart disease |  |  |  |  |  |
| Model 1 | 0.98 (0.86-1.11) | 1.12 (1.00-1.25) | 1.63 (1.47-1.81) | 1.76 (1.32-2.30) | 2.49 (2.22-2.79) |
| Model 2 | 0.99 (0.88-1.13) | 1.15 (1.03-1.28) | 1.68 (1.51-1.86) | 1.81 (1.36-2.37) | 2.53 (2.26-2.84) |
| Model 3 | 1.00 (0.88-1.13) | 1.14 (1.02-1.28) | 1.66 (1.50-1.85) | 1.76 (1.32-2.30) | 2.48 (2.21-2.80) |
| Myocardial infarction |  |  |  |  |  |
| Model 1 | 0.92 (0.78-1.08) | 1.12 (0.97-1.28) | 1.61 (1.41-1.83) | 1.61 (1.10-2.28) | 2.48 (2.15-2.86) |
| Model 2 | 0.94 (0.80-1.10) | 1.15 (1.00-1.32) | 1.66 (1.45-1.89) | 1.65 (1.13-2.34) | 2.53 (2.19-2.92) |
| Model 3 | 0.94 (0.80-1.11) | 1.14 (0.99-1.31) | 1.65 (1.44-1.88) | 1.58 (1.08-2.24) | 2.45 (2.11-2.84) |
| Cerebrovascular disease |  |  |  |  |  |
| Model 1 | 1.15 (1.02-1.30) | 1.45 (1.31-1.62) | 2.39 (2.17-2.64) | 2.39 (1.85-3.04) | 5.33 (4.83-5.88) |
| Model 2 | 1.18 (1.04-1.33) | 1.49 (1.34-1.65) | 2.44 (2.21-2.70) | 2.43 (1.88-3.09) | 5.35 (4.85-5.92) |
| Model 3 | 1.20 (1.06-1.36) | 1.53 (1.37-1.70) | 2.52 (2.28-2.78) | 2.51 (1.94-3.21) | 5.60 (5.06-6.21) |
| Hemorrhagic stroke |  |  |  |  |  |
| Model 1 | 1.21 (1.03-1.42) | 1.61 (1.40-1.86) | 2.75 (2.41-3.14) | 2.73 (1.97-3.69) | 6.58 (5.79-7.50) |
| Model 2 | 1.23 (1.04-1.45) | 1.64 (1.42-1.89) | 2.79 (2.45-3.19) | 2.75 (1.98-3.72) | 6.57 (5.77-7.50) |
| Model 3 | 1.26 (1.07-1.48) | 1.69 (1.47-1.95) | 2.90 (2.53-3.32) | 2.86 (2.06-3.88) | 6.91 (6.05-7.92) |
| Ischemic stroke |  |  |  |  |  |
| Model 1 | 0.97 (0.74-1.27) | 1.32 (1.05-1.66) | 1.94 (1.58-2.41) | 2.02 (1.08-3.45) | 3.74 (3.00-4.68) |
| Model 2 | 0.99 (0.76-1.30) | 1.35 (1.07-1.70) | 1.98 (1.61-2.47) | 2.05 (1.10-3.50) | 3.75 (2.99-4.70) |
| Model 3 | 1.01 (0.77-1.33) | 1.38 (1.10-1.75) | 2.04 (1.65-2.55) | 2.13 (1.14-3.65) | 3.93 (3.12-4.97) |

Abbreviations: ISH, isolated systolic hypertension; IDH, isolated diastolic hypertension; SDH, systolic-diastolic hypertension.

Reference: Normal blood pressure.

^a^ Model 1: adjustment for age at risk; Model 2: further adjustment for level of education, marital status, smoking status, alcohol consumption, intake frequencies of red meat, fresh fruits, and vegetables, MET, and survey seasons; Model 3: further adjustment for prevalent diabetes at baseline, family medical history (only adjusted for in corresponding analysis of cause-specific mortality), BMI, and heart rate. Stratified according to five-year age group, sex, and survey sites. Normal BP is the reference.
