# Supplementary material for: Association between blood pressure categories and cardiovascular disease mortality in China
Source: PLoS One. 2021 Jul 30;16(7):e0255373. doi: 10.1371/journal.pone.0255373 (PMC8323908; doi:10.1371/journal.pone.0255373)
Supplement: S4 Table — (DOCX) [file pone.0255373.s007.docx]

**S4 Table. Associations of prehypertension and hypertension subtypes with mortality from cardiovascular diseases and its major subtypes by sex ^a^**

| **Cause of death** | **Prehypertension-low** | **Prehypertension-high** | **Hypertension** | | |
| --- | --- | --- | --- | --- | --- |
|  |  |  | **ISH** | **IDH** | **SDH** |
| **Male** |  |  |  |  |  |
| No. of participants | 30 193 | 49 631 | 25 574 | 4593 | 20 243 |
| No. of person-years | 300 827 | 492 063 | 244 558 | 46 042 | 195 569 |
| Cardiovascular disease |  |  |  |  |  |
| No. of deaths | 563 | 1097 | 1664 | 89 | 1328 |
| Incidence rate (no./1,000 person-y) | 1.87 | 2.23 | 6.80 | 1.93 | 6.79 |
| HR (95%CI) | 1.03 (0.92-1.15) | 1.37 (1.25-1.51) | 2.08 (1.90-2.28) | 1.90 (1.51-2.35) | 3.69 (3.36-4.06) |
| Ischemic heart disease |  |  |  |  |  |
| No. of deaths | 234 | 424 | 594 | 36 | 392 |
| Incidence rate (no./1,000 person-y) | 0.78 | 0.86 | 2.43 | 0.78 | 2.00 |
| HR (95%CI) | 0.92 (0.78-1.09) | 1.15 (0.99-1.33) | 1.62 (1.41-1.87) | 1.55 (1.08-2.16) | 2.40 (2.06-2.80) |
| Myocardial infarction |  |  |  |  |  |
| No. of deaths | 148 | 272 | 364 | 23 | 245 |
| Incidence rate (no./1,000 person-y) | 0.49 | 0.55 | 1.49 | 0.50 | 1.25 |
| HR (95%CI) | 0.89 (0.72-1.09) | 1.13 (0.94-1.35) | 1.57 (1.32-1.88) | 1.57 (0.99-2.37) | 2.29 (1.89-2.78) |
| Cerebrovascular disease |  |  |  |  |  |
| No. of deaths | 264 | 558 | 918 | 44 | 856 |
| Incidence rate (no./1,000 person-y) | 0.88 | 1.13 | 3.75 | 0.96 | 4.38 |
| HR (95%CI) | 1.12 (0.95-1.31) | 1.62 (1.41-1.86) | 2.64 (2.31-3.02) | 2.32 (1.66-3.15) | 5.46 (4.78-6.26) |
| Hemorrhagic stroke |  |  |  |  |  |
| No. of deaths | 145 | 338 | 535 | 28 | 571 |
| Incidence rate (no./1,000 person-y) | 0.48 | 0.69 | 2.19 | 0.61 | 2.92 |
| HR (95%CI) | 1.14 (0.91-1.41) | 1.81 (1.51-2.18) | 3.03 (2.55-3.63) | 2.59 (1.69-3.80) | 6.77 (5.68-8.09) |
| Ischemic stroke |  |  |  |  |  |
| No. of deaths | 56 | 112 | 179 | 8 | 131 |
| Incidence rate (no./1,000 person-y) | 0.19 | 0.23 | 0.73 | 0.17 | 0.67 |
| HR (95%CI) | 1.08 (0.76-1.53) | 1.49 (1.10-2.02) | 2.23 (1.68-2.99) | 1.87 (0.82-3.67) | 3.79 (2.80-5.15) |
| **Female** |  |  |  |  |  |
| No. of participants | 39 937 | 58 329 | 35 134 | 3794 | 18 784 |
| No. of person-years | 404 483 | 590 909 | 349 355 | 39 014 | 188 215 |
| Cardiovascular disease |  |  |  |  |  |
| No. of deaths | 476 | 690 | 1469 | 59 | 895 |
| Incidence rate (no./1,000 person-y) | 1.18 | 1.17 | 4.20 | 1.51 | 4.76 |
| HR (95%CI) | 1.20 (1.06-1.36) | 1.25 (1.12-1.41) | 2.01 (1.81-2.23) | 2.90 (2.19-3.76) | 4.01 (3.58-4.49) |
| Ischemic heart disease |  |  |  |  |  |
| No. of deaths | 189 | 267 | 565 | 20 | 257 |
| Incidence rate (no./1,000 person-y) | 0.47 | 0.45 | 1.62 | 0.51 | 1.37 |
| HR (95%CI) | 1.13 (0.93-1.37) | 1.14 (0.95-1.37) | 1.75 (1.48-2.06) | 2.29 (1.40-3.53) | 2.63 (2.18-3.18) |
| Myocardial infarction |  |  |  |  |  |
| No. of deaths | 110 | 172 | 349 | 9 | 178 |
| Incidence rate (no./1,000 person-y) | 0.27 | 0.29 | 1.00 | 0.23 | 0.95 |
| HR (95%CI) | 1.03 (0.80-1.33) | 1.15 (0.92-1.44) | 1.76 (1.43-2.17) | 1.58 (0.75-2.93) | 2.71 (2.15-3.43) |
| Cerebrovascular disease |  |  |  |  |  |
| No. of deaths | 235 | 342 | 765 | 26 | 583 |
| Incidence rate (no./1,000 person-y) | 0.58 | 0.58 | 2.19 | 0.67 | 3.10 |
| HR (95%CI) | 1.32 (1.10-1.58) | 1.40 (1.19-1.66) | 2.39 (2.06-2.79) | 2.96 (1.93-4.36) | 5.88 (5.03-6.90) |
| Hemorrhagic stroke |  |  |  |  |  |
| No. of deaths | 134 | 192 | 427 | 17 | 390 |
| Incidence rate (no./1,000 person-y) | 0.33 | 0.32 | 1.22 | 0.44 | 2.07 |
| HR (95%CI) | 1.44 (1.13-1.83) | 1.52 (1.21-1.90) | 2.76 (2.25-3.40) | 3.49 (2.03-5.64) | 7.24 (5.89-8.96) |
| Ischemic stroke |  |  |  |  |  |
| No. of deaths | 37 | 69 | 152 | 5 | 90 |
| Incidence rate (no./1,000 person-y) | 0.09 | 0.12 | 0.44 | 0.13 | 0.48 |
| HR (95%CI) | 0.94 (0.61-1.43) | 1.25 (0.87-1.81) | 1.86 (1.34-2.61) | 2.82 (0.98-6.44) | 4.27 (2.99-6.16) |

Abbreviations: ISH, isolated systolic hypertension; IDH, isolated diastolic hypertension; SDH, systolic-diastolic hypertension; HR, hazard ratios; CI, confidence interval.

Reference: Normal blood pressure.

^a^ Multi-adjusted hazard ratios were adjusted for age, education level, marital status, smoking status, alcohol consumption, intake of vegetables, fruits, and red meat, physical activity, body mass index, survey season, heart rate, diabetes at baseline, family history of heart attack, stroke (only adjusted for in corresponding analysis of cause-specific mortality), menopausal status (only adjust for female) and were stratified according to five-year age group and survey sites. Statistically significant heterogeneity was observed in the associations between blood pressure categories and overall CVD, ischemic heart disease, cerebrovascular diseases and hemorrhagic stroke across sex (*P* values for interaction < .05), but not for myocardial infarction (*P* = .15) and ischemic stroke (*P* = .81).
