# Supplementary material for: Association between blood pressure categories and cardiovascular disease mortality in China
Source: PLoS One. 2021 Jul 30;16(7):e0255373. doi: 10.1371/journal.pone.0255373 (PMC8323908; doi:10.1371/journal.pone.0255373)
Supplement: S5 Table — (DOCX) [file pone.0255373.s008.docx]

**S5 Table. Associations of prehypertension and hypertension subtypes with mortality from cardiovascular diseases by survey sites ^a^**

| **Survey site** | **No. of participants** | **Prehypertension-low** | **Prehypertension-high** | **ISH** | **IDH** | **SDH** |
| --- | --- | --- | --- | --- | --- | --- |
|  |  | **HR (95%CI)** | **HR (95%CI)** | **HR (95%CI)** | **HR (95%CI)** | **HR (95%CI)** |
| Qingdao (Urban) | 29 226 | 0.87 (0.56-1.34) | 0.98 (0.68-1.43) | 1.41 (0.99-2.02) | 1.36 (0.62-2.66) | 2.15 (1.48-3.15) |
| Harbin (Urban) | 44 147 | 1.29 (1.00-1.65) | 1.62 (1.31-2.00) | 2.34 (1.89-2.91) | 1.81 (1.11-2.81) | 4.33 (3.47-5.41) |
| Haikou (Urban) | 26 166 | 1.04 (0.74-1.43) | 1.41 (1.06-1.87) | 1.41 (1.06-1.87) | 1.00 (0.25-2.68) | 2.80 (1.99-3.90) |
| Suzhou (Urban) | 43 265 | 1.05 (0.70-1.56) | 1.03 (0.72-1.49) | 1.75 (1.27-2.45) | 1.44 (0.50-3.31) | 2.49 (1.73-3.61) |
| Liuzhou (Urban) | 39 980 | 1.52 (1.15-2.02) | 1.80 (1.39-2.36) | 2.62 (2.04-3.39) | 2.61 (1.16-5.08) | 5.27 (3.96-7.02) |
| Sichuan (Rural) | 52 516 | 0.99 (0.79-1.24) | 1.09 (0.89-1.33) | 1.64 (1.35-2.01) | 2.15 (1.27-3.42) | 3.55 (2.91-4.35) |
| Gansu (Rural) | 45 232 | 1.06 (0.86-1.30) | 1.34 (1.12-1.61) | 2.18 (1.85-2.57) | 2.46 (1.65-3.56) | 4.69 (3.98-5.53) |
| Henan (Rural) | 52 132 | 0.98 (0.77-1.24) | 1.30 (1.07-1.59) | 2.21 (1.84-2.67) | 2.97 (1.86-4.53) | 3.82 (3.15-4.66) |
| Zhejiang (Rural) | 47 047 | 1.57 (1.06-2.33) | 1.43 (1.00-2.07) | 2.57 (1.86-3.60) | 4.01 (2.07-7.26) | 3.49 (2.45-5.04) |
| Hunan (Rural) | 51 266 | 1.01 (0.83-1.21) | 1.29 (1.09-1.52) | 2.00 (1.72-2.34) | 2.15 (1.28-3.38) | 3.67 (3.09-4.35) |

Abbreviations: ISH, isolated systolic hypertension; IDH, isolated diastolic hypertension; SDH, systolic-diastolic hypertension; HR, hazard ratios; CI, confidence interval.

Reference: Normal blood pressure.

^a^ Adjusted for age, education level, marital status, smoking status, alcohol consumption, intake of vegetables, fruits, and red meat, physical activity, body mass index, survey season, heart rate, diabetes at baseline, and family history of cardiovascular disease, and were stratified according to five-year age group and sex.
