# Supplementary material for: Association between blood pressure categories and cardiovascular disease mortality in China
Source: PLoS One. 2021 Jul 30;16(7):e0255373. doi: 10.1371/journal.pone.0255373 (PMC8323908; doi:10.1371/journal.pone.0255373)
Supplement: S6 Table — (DOCX) [file pone.0255373.s009.docx]

**S6 Table. Associations of blood pressure categories with deaths due to cardiovascular diseases among participants excluding the first two years of follow-up ^a^**

| **Cause of death** | **Prehypertension-low** | **Prehypertension-high** | **Hypertension** | | |
| --- | --- | --- | --- | --- | --- |
|  |  |  | **ISH** | **IDH** | **SDH** |
| No. of participants | 69 641 | 107 169 | 59 858 | 8326 | 38 463 |
| No. of person-years | 565 484 | 867 739 | 473 203 | 68 335 | 306 237 |
| Cardiovascular disease |  |  |  |  |  |
| No. of deaths | 940 | 1626 | 2851 | 127 | 1977 |
| HR (95%CI) | 1.11 (1.02-1.21) | 1.35 (1.25-1.46) | 2.08 (1.93-2.23) | 2.14 (1.77-2.57) | 3.84 (3.55-4.14) |
| Ischemic heart disease |  |  |  |  |  |
| No. of deaths | 384 | 626 | 1069 | 47 | 592 |
| HR (95%CI) | 1.01 (0.88-1.15) | 1.16 (1.03-1.30) | 1.71 (1.53-1.92) | 1.68 (1.22-2.24) | 2.56 (2.26-2.90) |
| Myocardial infarction |  |  |  |  |  |
| No. of deaths | 234 | 398 | 657 | 27 | 377 |
| HR (95%CI) | 0.97 (0.81-1.14) | 1.15 (0.995-1.34) | 1.72 (1.49-1.98) | 1.54 (1.02-2.25) | 2.51 (2.15-2.94) |
| Cerebrovascular disease |  |  |  |  |  |
| No. of deaths | 452 | 825 | 1518 | 63 | 1262 |
| HR (95%CI) | 1.21 (1.07-1.38) | 1.56 (1.40-1.75) | 2.52 (2.27-2.81) | 2.55 (1.94-3.30) | 5.55 (4.98-6.19) |
| Hemorrhagic stroke |  |  |  |  |  |
| No. of deaths | 246 | 472 | 840 | 40 | 819 |
| HR (95%CI) | 1.28 (1.08-1.53) | 1.74 (1.50-2.03) | 2.92 (2.53-3.39) | 2.99 (2.11-4.12) | 6.94 (6.01-8.03) |
| Ischemic stroke |  |  |  |  |  |
| No. of deaths | 92 | 170 | 304 | 12 | 200 |
| HR (95%CI) | 1.13 (0.85-1.49) | 1.46 (1.15-1.87) | 2.12 (1.69-2.68) | 2.20 (1.14-3.85) | 4.01 (3.14-5.14) |

Abbreviations: ISH, isolated systolic hypertension; IDH, isolated diastolic hypertension; SDH, systolic-diastolic hypertension; HR, hazard ratios; CI, confidence interval.

Reference: Normal blood pressure.

^a^ Multi-adjusted hazard ratios were adjusted for age, education level, marital status, smoking status, alcohol consumption, intake of vegetables, fruits, and red meat, physical activity, body mass index, survey season, heart rate, diabetes at baseline, family history of heart attack, stroke (only adjusted for in corresponding analysis of cause-specific mortality) and were stratified according to five-year age group, sex, and survey sites.
