# Supplementary material for: Association between blood pressure categories and cardiovascular disease mortality in China
Source: PLoS One. 2021 Jul 30;16(7):e0255373. doi: 10.1371/journal.pone.0255373 (PMC8323908; doi:10.1371/journal.pone.0255373)
Supplement: S1 Text — (DOCX) [file pone.0255373.s011.docx]

**S1 TEXT. Baseline questionnaire in the China Kadoorie Biobank study**

https://www.ckbiobank.org/site/binaries/content/assets/resources/pdf/qs_baseline-final-from10june2004.pdf
