# Supplementary material for: Association between blood pressure categories and cardiovascular disease mortality in China
Source: PLoS One. 2021 Jul 30;16(7):e0255373. doi: 10.1371/journal.pone.0255373 (PMC8323908; doi:10.1371/journal.pone.0255373)
Supplement: Attachment — Submitted filename: reviewer .docx [file pone.0255373.s012.docx]

This study, involving 0.4 million people, investigated the association between hypertension subtypes and CVD outcomes. It was well written.

There are several comments that might be taken into consideration.

1. It would be better to demonstrated the “inclusion criteria” and “exclusion criteria” of the CKB study.

2. For better understanding, it is recommended to use the definition of hypertension grades according to the 2018 ESC guidelines of hypertension as “optimal, normal, high normal”, rather than using the terms as “normal, prehypertension-low, prehypertension-high”.

3. Please state why patients with antihypertension treatment at baseline were excluded.

4. Please state the proportion of “loss to follow-up” and how it was addressed.
