# Supplementary material for: Association between blood pressure categories and cardiovascular disease mortality in China
Source: PLoS One. 2021 Jul 30;16(7):e0255373. doi: 10.1371/journal.pone.0255373 (PMC8323908; doi:10.1371/journal.pone.0255373)
Supplement: Attachment — Submitted filename: Second round review.docx [file pone.0255373.s014.docx]

There are two minor comments which need to be addressed.

1. It would cause selection bias if those patients with antihypertensive agents were excluded. In the current study, antihypertensive treatment could be an unmeasured covariate which would affect the results. It should be further discussed in the part of Discussion.

.2. It’s a little bit confusing that the author defined “prehypertension-high” as “hypertension stage 1” according to ACC/AHA guideline, while the definitions of IDH and ISH were not consist with ACC/AHA guideline. It should have a much clearer expression.
