# Supplementary material for: Association between blood pressure categories and cardiovascular disease mortality in China
Source: PLoS One. 2021 Jul 30;16(7):e0255373. doi: 10.1371/journal.pone.0255373 (PMC8323908; doi:10.1371/journal.pone.0255373)
Supplement: Attachment — Submitted filename: Response to Reviewers.docx [file pone.0255373.s015.docx]

**There are two minor comments which need to be addressed.**

We thank the reviewer for the valuable comments. All comments and suggestions have been carefully addressed in the revised manuscript. Details of the comments are responded to point-by-point as follows.

**1. It would cause selection bias if those patients with antihypertensive agents were excluded. In the current study, antihypertensive treatment could be an unmeasured covariate which would affect the results. It should be further discussed in the part of Discussion.**

We thank the reviewer's comments. We agree that excluding patients with antihypertension agents might cause selection bias given that hypertension with treatment might be more aware of their health status and more accessible to health care or might have severe symptom due to high blood pressure compared to those without treatment, which could affect future CVD risk. However, taking antihypertensive medicines would change the level of blood pressure and lead to a misclassification of hypertension subtypes. Given that it is not possible to know their blood pressure level before taking antihypertensive medicines, to exclude participants with antihypertensive medicines is more reasonable for the current study. Thus, our findings could be generalized to the population without taking antihypertensive medicines.

We added the corresponding issue in the limitations section of discussion (page 21, lines 359-364).

"Second, we excluded participants who taking antihypertensive medicines, which might cause selection bias and limit the extrapolation of our findings. However, antihypertensive medication would affect the patients' blood pressure level, leading to misclassification of BP categories. Hence, it is more reasonable to restrict study participants without antihypertensive treatment."

**2. It's a little bit confusing that the author defined "prehypertension-high" as "hypertension stage 1" according to ACC/AHA guideline, while the definitions of IDH and ISH were not consist with ACC/AHA guideline. It should have a much clearer expression.**

We thank the reviewer's comments. In the current study, we defined the blood pressure categories mainly referred to the JNC-7 guideline, which defined prehypertension and hypertension subtypes (i.e. ISH, IDH, and SDH). The definition was adopted in previous studies (*Arima H et al, Hypertension 2012; Kelly TN et al, Circulation 2008*). However, the prehypertension (i.e., SBP/DBP 120 to 139/80 to 89 mm Hg) had a broad range of blood pressure, and its effects on CVD risk were heterogeneous within prehypertension (*Y Huang et al, American Heart Journal 2014*). In addition, the 2017 ACC/AHA guideline newly defined the high-range blood pressure within prehypertension as hypertension. Thus, we combined the prehypertension definition in JNC-7 with the new cut-off in 2017 ACC/AHA guideline, i.e., SBP 120−129 and DBP <80 mm Hg; SBP 130−139 and/or DBP 80−89 mm Hg. We revised the manuscript (page 7, lines 127-132) to clarify our categorisation.

"According to the JNC-7, BP categories were defined into five groups 1) normal (SBP <120 and DBP <80 mm Hg); 2) prehypertension (SBP 120−139 and/or DBP 80−89 mm Hg); 3) ISH (SBP ≥140 and DBP <90 mm Hg); 4) IDH (SBP <140 and DBP ≥90 mm Hg); 5) SDH (SBP ≥140 and DBP ≥90 mm Hg) (*Chobanian A V., Hypertension 2003*). In the 2017 ACC/AHA hypertension guideline, hypertension was defined as SBP ≥130 mmHg and/or DPB ≥90 mmHg (*Whelton PK, Hypertension 2018*). To estimate the effect of "Elevated" and "Hypertension stage 1", we further divided prehypertension into prehypertension-low (equal to "Elevated", SBP 120−129 and DBP <80 mm Hg) and prehypertension-high (equal to "Hypertension stage 1", SBP 130−139 and/or DBP 80−89 mm Hg) (*Whelton PK, Hypertension 2018*)."

**Reviewer's Responses to Questions**

**Have the authors made all data underlying the findings in their manuscript fully available?**

The PLOS Data policy requires authors to make all data underlying the findings described in their manuscript fully available without restriction, with rare exception (please refer to the Data Availability Statement in the manuscript PDF file). The data should be provided as part of the manuscript or its supporting information, or deposited to a public repository. For example, in addition to summary statistics, the data points behind means, medians and variance measures should be available. If there are restrictions on publicly sharing data—e.g. participant privacy or use of data from a third party—those must be specified.

Reviewer #1: (No Response)

Reviewer #2: Yes

Reviewer #3: No

For the data availability, we want to state as follows.

"According to the Regulation of the People's Republic of China on the Administration of Human Genetic Resources, we are not allowed to provide Chinese human clinical and genetic data abroad without official approval so the data in the present study cannot be shared without restrictions. However, researchers who are interested in accessing and analyzing data collected in the China Kadoorie Biobank (CKB) study may contact the data use and access committee ([**http://www.ckbiobank.org/site/Research/Data+Access+Policy**](http://www.ckbiobank.org/site/Research/Data+Access+Policy)). As stated in the policy, as data custodian, the CKB study group must maintain the integrity of the database for future use and regulate data access to comply with prior conditions agreed with the Chinese government."
